# Microglia as a Hub for Suicide Neuropathology: Future Investigation and Prevention Targets

**DOI:** 10.3389/fncel.2022.839396

**Published:** 2022-05-18

**Authors:** Elisa Gonçalves de Andrade, Fernando González Ibáñez, Marie-Ève Tremblay

**Affiliations:** ^1^Neuroscience Graduate Program, Division of Medical Sciences, University of Victoria, Victoria, BC, Canada; ^2^Division of Medical Sciences, University of Victoria, Victoria, BC, Canada; ^3^Axe Neurosciences, Centre de Recherche du CHU de Québec, Université Laval, Québec City, QC, Canada; ^4^Department of Neurology and Neurosurgery, McGill University, Montréal, QC, Canada; ^5^Department of Molecular Medicine, Université Laval, Québec City, QC, Canada; ^6^Department of Biochemistry and Molecular Biology, University of British Columbia, Vancouver, BC, Canada; ^7^Centre for Advanced Materials and Related Technology (CAMTEC), University of Victoria, Victoria, BC, Canada

**Keywords:** microglia, suicide, stress, epigenetics, inflammation, oxidative stress, neuronal support, synaptic plasticity

## Abstract

Suicide is a complex public health challenge associated worldwide with one death every 40 s. Research advances in the neuropathology of suicidal behaviors (SB) have defined discrete brain changes which may hold the key to suicide prevention. Physiological differences in microglia, the resident immune cells of the brain, are present in post-mortem tissue samples of individuals who died by suicide. Furthermore, microglia are mechanistically implicated in the outcomes of important risk factors for SB, including early-life adversity, stressful life events, and psychiatric disorders. SB risk factors result in inflammatory and oxidative stress activities which could converge to microglial synaptic remodeling affecting susceptibility or resistance to SB. To push further this perspective, in this Review we summarize current areas of opportunity that could untangle the functional participation of microglia in the context of suicide. Our discussion centers around microglial state diversity in respect to morphology, gene and protein expression, as well as function, depending on various factors, namely brain region, age, and sex.

## Introduction

Suicide is a complex behavior resulting from intricate, multi-dimensional interactions between various social, cultural, biological, psychological, and environmental factors (Turecki et al., 2019). Worldwide, suicide is the second and third leading cause of premature death in individuals between 15–29 and 15–44 years of age, respectively (Bachmann, 2018). While the prevalence may vary according to the country, suicide deaths are more common in males, whereas suicide attempts are more frequent in females (Bachmann, 2018). Elevated suicide rates in males could be connected to a preferential use of more lethal methods compared to females, in addition to important differences in socialization between sexes (Tsirigotis et al., 2011). For instance, traditional masculine role norms include self-reliance which may negatively impact their capacity to seek help (Seidler et al., 2021). Beyond age and sex, another crucial risk factor for suicide is the presence of psychiatric disorders such as MDD and SCZ (see Table 1 for full definitions of abbreviations), which are associated with at least 43.2 and 9.2% of suicide deaths, respectively, in North America (Arsenault-Lapierre et al., 2004). Although the categorization of SB is subject to debate (Goodfellow et al., 2019), in this Review we use this term to refer to suicide, suicide attempts and preparatory acts (Nock, 2014), while suicidal ideation is used to specify active or passive thoughts about ending one’s own life (Turecki et al., 2019).

**TABLE 1:** Full list of abbreviations and corresponding definitions.

Abbreviation	Definition
8-OHdG	8-hydroxy-2′-deoxyguanosine
ACC	Anterior cingulate cortex
AD	Alzheimer’s disease
aMCC	Anterior midcingulate cortex
AMY	Amygdala
ATP	Adenosine triphosphate
BAM	Border-associated macrophages
BBB	Blood-brain barrier
BDNF	Brain-derived neurotrophic factor
BLA	Basolateral amygdala
BrdU	Bromodeoxyuridine
C1q	Complement C1q
CA	*Cornu ammonis*
CCL2, MCP-1	C-C motif chemokine ligand 2
CCR2	C-C chemokine receptor 2
CD11b	Integrin subunit alpha M
CD163	CD163 molecule
CD45	Tyrosine phosphatase receptor type C
CD68	CD68 molecule
CLDN5	Claudin 5
CNS	Central nervous system
CRP	C-reactive protein
CRS	Chronic restraint stress
CSDS	Chronic social defeat stress
CSF	Cerebrospinal fluid
CSF1R	Colony-stimulating factor 1 receptor
CUMS	Chronic unpredictable mild stress
CUS	Chronic unpredictable stress
CX3CL1	C-X3-C motif chemokine ligand 1
CX3CR1	C-X3-C motif chemokine receptor 1
dACC	Dorsal ACC
DCX	Doublecortin
DG	Dentate gyrus
DLPFC	Dorsolateral prefrontal cortex
DLS	Dorsolateral striatum
DM	Dark microglia
DPFWM	Dorsal prefrontal white matter
DRN	Dorsal raphe nucleus
ELA	Early-life adversity
ESI	Early-life social isolation
ESS	Early-life social stress
fMRI	Functional magnetic resonance imaging
FOSB	FBJ osteosarcoma oncogene B
GABA	Cortical gamma-aminobutyric acid
GC	Glucocorticoids
HEXB	Hexosaminidase
HIP	Hippocampus
HLA-DR	Major histocompatibility complex, class II, DR
HPA	Hypothalamus–pituitary–adrenal
HYP	Hypothalamus
IBA1	Ionized calcium-binding adapter molecule 1
IDO	Indoleamine 2,3-dioxygenase
IFN	Interferon
IL	Interleukin
IL1R1	Interleukin 1 receptor type 1
Ki67	Nuclear protein Ki67
LPS	Lipopolysaccharide
MAC387	S100 calcium binding protein A9
MDD	Major depressive disorder
MFG	Medial frontal gyrus
MIA	Maternal immune activation
MTN	Mediodorsal thalamic nucleus
NA	*Nucleus accumbens*
NFκB	Nuclear factor of kappa light polypeptide gene enhancer in B cells
NMDAR	N-methyl-D-aspartic acid receptor
NOX2	Nicotinamide adenine dinucleotide phosphate oxidase
P2RY12	P2Y purinoceptor 12
PET	Positron emission tomography
PFC	Prefrontal cortex
PMI	Post-mortem interval
QUIN	Quinolinic acid
ROS	Reactive oxygen species
SALL1	Spalt like transcription factor 1
SB	Suicidal behaviors
SCZ	Schizophrenia
STG	Superior temporal gyrus
SZ	Subventricular zone
TBR2	Eomes
TDO	Tryptophan 2,3-dioxygenase
TLM	Thalamus
TLR	Toll-like receptor
TMEM119	Transmembrane protein 119
TNF	Tumor necrosis factor
TREM2	Triggering receptor expressed on myeloid cells 2
TSPO	Translocator protein
VLPFC	Ventrolateral prefrontal cortex
VPFWM	Ventral prefrontal white matter

In the hope of identifying novel therapeutic targets to prevent SB, research has looked for changes in the CNS that could hint at the biological alterations responsible for SB and their risk factors. Studies of human brain samples from individuals who died by suicide have revealed direct changes in microglial numbers, morphology, as well as gene and protein expression (see Supplementary Table 1), although their mechanisms of action remain unknown (Suzuki et al., 2019; Baharikhoob and Kolla, 2020). Microglia are the resident immune cells of the CNS (Šimončičová et al., 2022). They perform classic immune roles involving debris clearance (Lampron et al., 2015), phagocytosis of infected or apoptotic cells (Sierra et al., 2010; Györffy et al., 2018) and production of immune mediators (Aloisi, 2001). Their function, however, goes beyond immunity (Šimončičová et al., 2022). Notably, microglia contribute to the development, maintenance, and plasticity of the CNS. These cells modulate the survival of newborn neurons and stimulate the formation of dendritic spines via neurotrophic support (Sierra et al., 2010; Parkhurst et al., 2013). In addition to contributing to synapse formation (Miyamoto et al., 2016), microglia participate in synaptic remodeling through the stripping of pre- and post-synaptic elements (Trapp et al., 2007), pruning of synapses via partial (Weinhard et al., 2018) or full phagocytosis (Linnartz et al., 2012; Schafer et al., 2012; Györffy et al., 2018), and remodeling of the extracellular matrix (Nguyen et al., 2020). Furthermore, microglia assist astrocytic cellular maturation and responses to immune insults (Vainchtein and Molofsky, 2020). Similarly, microglia are needed for the maturation of oligodendrocyte progenitors (Hagemeyer et al., 2017), myelination of axons (Hughes and Appel, 2020), vascular formation and remodeling, as well as homeostatic BBB permeability regulation (Joost et al., 2019). By fine-tuning CNS connectivity and cellular signaling, microglia influence behaviors involved in learning, memory, and sociability both in health and disease (Tay et al., 2018; Bordeleau et al., 2019; Tremblay, 2021).

**FIGURE 1:**
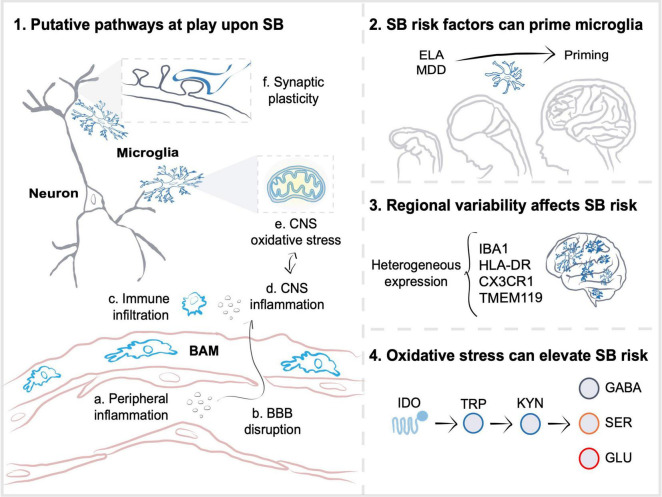
Microglia participate in pathways altered upon suicidal behaviors. (1) As a result of stress-susceptibility, inflammation in the periphery (a) can contribute to blood-brain barrier (BBB) disruption (b) and infiltration of peripheral cells or border-associated macrophages (BAM) (c) to the central nervous system (CNS) parenchyma. Stress-induced inflammatory (d) and oxidative stress (e) molecules, such as cytokines, reactive oxygen species or adenosine triphosphate, work in a positive feedback loop that could affect microglial synaptic plasticity regulation (f), and increase suicidal behaviors (SB) risk. (2) SB risk factors, including distal early-life adversity (ELA) and proximal major depressive disorder (MDD), are associated with microglial priming, in which elevated expression of genes related to phagocytosis, cellular proliferation, and vesicular release result in exacerbated inflammatory responses upon exposure to subsequent challenges, as well as to impaired synaptic development and function. (3) The regional diversity of microglia possibly participates in determining susceptibility to inflammation induced by SB risk factors. Region-specific differences in gene or protein expression of ionized calcium-binding adapter molecule 1 (IBA1), major histocompatibility complex class II, DR (HLA-DR), CX3C chemokine receptor 1 (CX3CR1), transmembrane protein 119 (TMEM119), as well as in density are observed in post-mortem CNS samples from individuals who died by suicide. (4) Similarly, distinct CNS areas likely have different oxidative stress responses to inflammation. Oxidative stress induced by inflammation is thought to affect mitochondrial and tryptophan metabolism, which may lead to an increased activity of microglial indoleamine 2,3-dioxygenase 1 (IDO), an enzyme that breaks tryptophan (TRP) down into kynurenine (KYN). Increased KYN to TRP ratio could decrease serotonin (SER) and gamma-aminobutyric acid (GABA) signaling, whilst elevating glutamate (GLU) excitotoxity in individuals with SB. Altogether, the multiple synaptic plasticity-related pathways implicate microglia in SB, warranting further investigation.

As microglia are intimately tuned to their microenvironment, the periphery and other environmental influences, these cells are likely responsive to the different SB risk factors (Turecki et al., 2014; Réus et al., 2015; Mondelli et al., 2017; Tay et al., 2018). Suicide risk factors add up across the lifespan and can be categorized according to their temporal distance to the SB’s onset (Turecki et al., 2019). They include distal factors such as ELA and genetic history; developmental factors linked to personality traits or cognitive deficits; and proximal factors comprising stressful life events and psychiatric disorders, such as MDD or SCZ (Turecki et al., 2019). Each one of these factors is further dependent on the individuals’ societal, demographic and economic circumstances (Bachmann, 2018; Turecki et al., 2019). Notably, the biological consequences of microglial responses to SB risk factors are as diverse and complex as suicide etiology itself (Underwood and Arango, 2011). Hence, in this Review, we focus on putative microglial pathways that can help explain why distal and proximal stress may translate into SB risk, taking into account the previous findings and the context under investigation, such as age, sex, and CNS region. In particular, we outline the outcomes connected to inflammation, oxidative stress and trophic balance (see Figure 1), increasingly associated with MDD, SCZ and SB risk (Suzuki et al., 2019; Baharikhoob and Kolla, 2020). We hope our considerations can open venues of future investigation that will uncover targets able to support suicide prevention.

## Suicidal Behaviors Are Associated With Increased Peripheral and Central Inflammatory Molecules

Stress is a physiological response to a variety of biological and psychological challenges (Lucassen et al., 2014). The physiological and cognitive outcomes of stress vary between susceptible and resistant individuals, influenced by their genetic, epigenetic, and behavioral characteristics, together with the duration and type of stressor (Russo et al., 2012; Dantzer et al., 2018). In vulnerable groups, stress is notably marked by unfavorable inflammatory responses (Ménard et al., 2017), which could help explain why distal or proximal stress increases SB risk (Suzuki et al., 2019; Baharikhoob and Kolla, 2020). Meta-analyses often find enhanced circulating protein levels of CRP and pro-inflammatory cytokines, such as IL6 and TNF, as well as reduced circulating protein levels of anti-inflammatory IL2 in individuals with SB (Serafini et al., 2020). Clinical studies indicate a relationship between enhanced plasma protein levels of IL6 and CRP with traits of aggression and impulsivity (Brodsky et al., 2001; Coccaro, 2006; Coccaro et al., 2014), frequently comorbid with psychiatric disorders and SB (Brundin et al., 2017). Moreover, IFNα therapy, regularly used to treat tumor malignancy and chronic viral hepatitis, is commonly associated with the development of SB (Brundin et al., 2017; Serafini et al., 2020). Despite the link between peripheral inflammation and SB (Figure 1), conflicting changes in brain expression of cytokines such as TNF, IL1β, IL6, and IL10 were reported across post-mortem studies of individuals who died by suicide (Supplementary Table 1; Torres-Platas et al., 2014b; Clark et al., 2016; Schiavone et al., 2016; Wang et al., 2018; Snijders et al., 2020). Furthermore, according to gene ontology analysis, the DLPFC, AMY, and TLM of individuals with MDD who died by suicide can present lower expression of gene sets related to microglial immune functions such as “chemokine receptor binding” and “cellular response to LPS” compared to age- and sex-matched individuals with MDD deceased from other causes or healthy controls (Pantazatos et al., 2017; Glavan et al., 2021). Such differences could be reconciled by the largely distinct brain regions studied, such as the VLPFC, ACC, and TLM, which likely present diverse baseline inter-individual immune activity, and therefore, a different susceptibility to peripheral inflammation (Hodes et al., 2014; Wood et al., 2015).

### Risk of Suicidal Behaviors After Stress May Involve the Inflammatory Activity of Microglia

Although more studies are warranted, there is *in vivo* indication that increased microglial inflammatory activity is present in individuals with SB. TSPO is an outer mitochondrial membrane protein expressed by microglia, macrophages, astrocytes, endothelial and ependymal cells in the brain, which shows a marked upregulation during CNS inflammation, as detected by PET imaging (Nutma et al., 2019; Pannell et al., 2020). Enhanced TSPO binding is observed, notably among the PFC, ACC, and insula, in medication-free patients with MDD actively experiencing a moderate-to-severe major MDD episode (Setiawan et al., 2015). Similarly, significantly greater TSPO binding is found in the ACC and insula of patients with MDD experiencing suicidal ideation compared to patients without suicidal thoughts (Holmes et al., 2018; Supplementary Table 1). In addition to *in vivo* studies, increased mRNA *Tspo* expression was detected in the DLPFC but not ACC of individuals with MDD irrespective of the cause of death, compared to age and PMI-matched controls (Zhang L. et al., 2021). Moreover, elevated protein expression, but not mRNA levels (Zhang L. et al., 2021), of HLA-DR were measured in various post-mortem areas, including the ACC, MTN, DLPFC, and DPFWM (Steiner et al., 2006, 2008; Torres-Platas et al., 2014b). HLA-DR is expressed by microglia, peripheral macrophages and BAM in the CNS (Steiner et al., 2006, 2008; Swanson et al., 2020; Prinz et al., 2021). In the periphery, HLA-DR is associated with antigen-presentation, however, within the CNS, although frequently upregulated in pathological contexts, its function remains unknown (Wolf et al., 2018). HLA-DR can be upregulated along CCL2 protein expression in white matter of male and female individuals with multiple sclerosis lesions (Peferoen et al., 2015). Therefore, a direct association of HLA-DR and pro-inflammatory microglial activity could be misleading (Walker and Lue, 2015). Prospective studies are encouraged to better characterize the immune response of microglia upon SB. Notably, the possible outcomes of the inflammatory activity of microglia are diverse and far-reaching in the CNS, as we outline next.

#### Stress-Induced Inflammation Could Engage Microglial Disruption of the Blood-Brain Barrier

Bidirectional interactions between the peripheral immune system and microglia are facilitated by cytokines crossing the BBB, primarily composed of endothelial cells, sealed together by tight junctions, covered by pericytes, astrocytic endfeet, and microglial processes (Bisht et al., 2016; Joost et al., 2019). A disrupted BBB can increase the influx of inflammatory molecules and cells to the CNS (Figure 1). Studies looking at CSF or serum concentrations of proteins typically found in the CNS, for instance, CSF hyaluronic acid (Ventorp et al., 2016) and albumin CSF/serum ratio (Bayard-Burfield et al., 1996), revealed that the BBB is compromised in individuals with SB and suicide ideation (Falcone et al., 2010; Torres-Platas et al., 2014b). Suggestive of decreased BBB integrity, mRNA downregulation of the tight junction protein *Cldn5* is present in the NA of individuals with MDD who died by suicide (Menard et al., 2017; Dudek et al., 2020). Compared to resistant and healthy controls, male mice susceptible to CSDS exhibited increased cortical leakage of intravenously injected dyes (Lehmann et al., 2018). Microglia isolated from the susceptible mice selectively expressed transcripts involved with extracellular matrix breakdown, inflammatory response, cytokine production, ROS and metabolic processes, which highly correlated with the transcriptomic changes associated with aging (Lehmann et al., 2018). Microglial stress susceptibility could be connected to the BBB disruption, requiring future investigation, particularly in older individuals, where this effect might be exacerbated and contribute to increasing SB risk. Indeed, robust data from animal models supports that aging is linked to microglial pro-inflammatory and oxidative stress activities, reduction in CNS surveillance, as well as appearance of microglial states, such as primed microglia (see section “Microglial Priming Is a Hallmark of Early-Life Adversity and May Contribute to Stress Susceptibility”), connected to synaptic dysfunction (Tay et al., 2018; Stratoulias et al., 2019). Correspondingly, although SB, in general, are more common at younger ages, suicide deaths rates are higher in older individuals in almost all countries (Conejero et al., 2018).

Alternatively, microglial resistance to stress could be associated with the protection of the BBB. Microglia were suggested to infiltrate the BBB toward the periphery and rescue its permeability via CLDN5 protein expression early on during systemic inflammation caused by daily LPS injections in adult male mice (Haruwaka et al., 2019). This protective role, however, changed at later time points, whereby microglia expressed higher levels of CD68 protein, a phagolysosomal activity marker present in human microglia/macrophages and associated with inflammation (Walker and Lue, 2015; Haruwaka et al., 2019). Elevated CD68 occurred along with an increased number of microglial inclusions of aquaporin 4, notably expressed in blood vessels and astrocytic endfeet, suggesting their phagocytosis (Haruwaka et al., 2019). Accordingly, CSDS in adult male mice increases the number of brain CD68-high cells, as revealed by flow cytometry (Lehmann et al., 2016). No significant differences in mRNA *Cd68* expression and CD68 immunostaining, however, were observed in the dACC and DPFWM or VPFWM, respectively, of individuals who died by suicide compared to PMI- (Torres-Platas et al., 2014b), sex-, psychiatric diagnosis- (Schnieder et al., 2014), and age-matched controls (Supplementary Table 1). Contrastingly, increased mRNA *Cd68* expression was detected in the DLPFC but not ACC of individuals with MDD who died by suicide, compared to age and PMI-matched controls (Zhang L. et al., 2021). Despite strong associations between stress, inflammation and phagocytosis, prospective studies are needed to understand if microglia play a detrimental or beneficial role in BBB integrity specifically in the context of SB.

##### Additional Mechanisms Can Impair the Blood-Brain Barrier After Stress-Induced Inflammation

Additional pathways mediated by stress susceptibility and increased inflammation can result in an impaired BBB permeability and elevated SB risk. Conditional knockdown of CLDN5 in NA blood vessels was enough to induce MDD-like behaviors and increase BBB permeability in CSDS-susceptible adult male mice (Menard et al., 2017). Moreover, it caused significant protein increase of IL6 NA levels, albeit not changing the density of NA CX3CR1-positive cells, largely microglia, but possibly representing peripheral macrophages and BAMs (Lehmann et al., 2016; Menard et al., 2017; Prinz et al., 2021). In a follow-up study, epigenetic regulation of CLDN5, detected also in samples of individuals with MDD who died by suicide, as well as changes in TNF/NFκB signaling, were connected to the observed susceptibility and resilience to stress in adult male mice (Dudek et al., 2020). Correspondingly, the NFκB inhibitor alpha was among the top ten genes implicated in the DLPFC individuals with MDD who died by suicide, compared to individuals with MDD who died of other causes and healthy controls (Zeng et al., 2020; Supplementary Table 1). Future studies are warranted to evaluate if and how microglia-dependent and -independent mechanisms of BBB disruption increase SB risk after stress.

##### Lifestyle Factors Such as Sleep Impact Microglial and Blood-Brain Barrier Function

BBB permeability appears to be similarly compromised by lifestyle factors, for instance, sleep. Chronic sleep restriction in adult male mice decreased mRNA expression of tight junction proteins, such as *Cldn5*, and increased cortical and subcortical uptake of intravenously injected dies, indicating lower BBB integrity (He et al., 2014). Circadian rhythms, which notably control sleep-wake patterns and are frequently disturbed in psychiatric disorders (Wulff et al., 2010), play a role in BBB regulation as well. According to studies in flies and mice, as well as in human *in vitro* models, BBB efflux mechanisms are stronger during the active or awake phases (Zhang et al., 2018; Zhang S. L. et al., 2021). Therefore, the BBB is not only physically restrictive, but also temporally restrictive (Cuddapah et al., 2019), indicating additional factors that can have a modulatory effect on the connection between peripheral and central immune systems. A multi-dimensional interaction between sleep, circadian rhythms, microglia and synaptic plasticity is suggested in the literature (Picard et al., 2021). Evidence from adult male rats support that circadian rhythms control the removal of synapses through complement opsonization (Choudhury et al., 2020). A meta-analysis looking at 42 studies published between 1982 and 2019 found a statistically significant but weak influence of sleep disturbances on SB (Harris et al., 2020). Despite the small effect, probably arising from inter-individual variability, lifestyle factors such as sleep are important elements to consider in the complex synergy of microglial features that convey susceptibility to psychiatric disorders (Picard et al., 2021) and SB (Glavan et al., 2021).

### Microglia Possibly Recruit Non-resident Central Nervous System Immune Cells Upon Stress Susceptibility

The CNS is populated by resident immune cells, microglia, but can be infiltrated by peripheral immune populations and BAM, such as macrophages from the choroid plexus, meninges and perivascular space (Prinz et al., 2021). Previous research in male adult mice has illustrated how infiltrating neutrophils are linked to inflammatory processes and MDD-like behavior (Aguilar-Valles et al., 2014), whereas monocytes in the CNS display signaling related to resolution and repair, as well as anxiety-like behavior (McKim et al., 2018; Liu et al., 2019). Studies on male mice exposed to CSDS found an increased recruitment of peripheral monocytes into the CNS in association with heightened anxiety- and MDD-like behaviors (Brevet et al., 2010; Wohleb et al., 2011, 2013, 2016; Weber et al., 2019; Yin et al., 2019; Picard et al., 2021). It is suggested, however, that infiltration results from wounding during CSDS. Additionally, in the study by Menard et al. (2017) CSDS caused an accumulation of peripheral CCR2-positive monocytes within blood vessels of the NA, without parenchymal infiltration.

Microglia are important for the recruitment of peripheral cells to the CNS. CSF1R inhibition, which reduces microglial numbers, prevented monocyte recruitment to the brain of adult male mice exposed to CSDS and abrogated anxiety-like symptoms, in a signaling cascade requiring microglial CCL2 and monocyte IL1β protein expression (McKim et al., 2018; Weber et al., 2019). According to studies in adult male mice, IL1R1, the IL1β receptor, is needed for monocyte recruitment to the ependymal and ventricular walls and for neutrophil infiltration through endothelial cells (Liu et al., 2019). IL1R1 expression in both routes of infiltration was linked to adaptations of microglia morphology (e.g., increased soma size in the DG) and protein expression (e.g., upregulated CD45) (Liu et al., 2019). By contrast, no difference in *Il1r1* mRNA expression was found in the dACC of individuals with MDD who died by suicide compared to age- and PMI-matched healthy controls (Torres-Platas et al., 2014b). While it remains unclear if similar IL1R1 mechanisms are active in SB, overall, these results indicate that the infiltration of immune cell populations likely results in a functional crosstalk with microglia that could be further investigated in the context of stress susceptibility and SB risk (Figure 1).

#### Conflicting Data Exists Around Immune Infiltration and Suicidal Behaviors

Despite the link between stress and CNS immune infiltration, previous post-mortem studies provide conflicting data on the presence of IBA1-positive cells associated with/within vessels, or on CD163-positive cell infiltration in the ACC, DPFWM and VPFWM of individuals who died by suicide compared to PMI- (Torres-Platas et al., 2014b), sex-, psychiatric diagnosis- (Schnieder et al., 2014, 2019), and age-matched controls. CD163 is a marker related to phagocytosis that is expressed by both human microglia and perivascular macrophages, although mainly in the latter (Swanson et al., 2020). CD163 protein expression was decreased in CD11b-positive microglia isolated from individuals with MDD compared to healthy controls, although without a specific effect on the one suicide death included in the study (Snijders et al., 2020). Moreover, elevated mRNA expression of *Ccl2*, alias *Mcp-1*, a chemokine involved in the recruitment of peripheral cells, and *Cd45* mRNA, usually enriched in peripheral macrophage populations, was found in the dACC of individuals with MDD who died by suicide compared to age- and PMI-matched healthy controls (Torres-Platas et al., 2014b). Contrastingly, it is important to consider that microglial populations can express high levels of CD45 protein in male mouse models of stroke and aging (Honarpisheh et al., 2020). Furthermore, decreased *Ccl2* transcripts were instead detected in the VLPFC of individuals with MDD compared to healthy controls, although lacking a suicide subgroup effect (Clark et al., 2016). Numerous factors could underlie the variable peripheral immune cell recruitment in SB, such as CNS regions, sexes, age, as well as inter-individual differences in stress susceptibility and resistance of the cohorts studied (Lehmann et al., 2018). Additionally, elevated CD163 immunostaining in the DPFWM was associated with a suicide-specific effect on microglial migration to abluminal surfaces of vessels, instead of peripheral immune cell infiltration (Schnieder et al., 2014, 2019). Previously reported elevations in IBA1-positive cells adjacent to vessels in the DPFWM of individuals who died by suicide compared to age-, sex- and psychiatric diagnoses-matched controls were not paralleled by changes in CD163-positive densities, indicating they likely arose from resident microglial staining (Schnieder et al., 2014, 2019). Increased association of microglia with vessels was shown upon systemic inflammation caused by daily LPS injections in male mice (Haruwaka et al., 2019) and a similar phenomenon might be present in SB. The dynamic interaction between microglia and blood vessels should be accounted for in future research looking at peripheral immune CNS invasion in SB.

#### Better Tools Are Required to Identify Specific Immune Contributions in Suicidal Behaviors

Microglia, perivascular macrophages and subdural leptomeningeal macrophages derive from yolk-sac erythromyeloid progenitors matured into myeloid precursor cells that seed the brain around E9.5 in mouse (Ginhoux et al., 2010; Kierdorf et al., 2013). Peripheral infiltrating monocytes or macrophages, by contrast, arise from erythromyeloid progenitors that travel to the fetal liver and later transition to the bone marrow starting around birth (Hoeffel et al., 2015; Stremmel et al., 2018). Similar ontogeny contributes to the large overlap in markers such as CX3CR1, CSF1R, CD11b, IBA1, HLA-DR, CD163, CD68, CD45 between microglia, BAM, and peripheral monocytes (Walker and Lue, 2015; Snijders et al., 2020; Swanson et al., 2020; Prinz et al., 2021). Region-specific analyses using largely microglia-specific markers such as TMEM119, P2RY12, SALL1, and HEXB (Prinz et al., 2021) are warranted to clarify the contribution of microglia *versus* non-microglial immune cells possibly acting together during SB.

### Microglial Priming Is a Hallmark of Early-Life Adversity and May Contribute to Stress Susceptibility

Stress-induced inflammation can prime microglia to respond more strongly when re-exposed to immune challenges (Neher and Cunningham, 2019). Based on a previous morphometric characterization of IBA1-positive cells in the dACC (Torres-Platas et al., 2014a,b) have suggested an increased density of primed microglia (bigger cell body, ellipsoid-like soma and fewer higher-order branches) in the dACC of individuals with MDD who died by suicide compared to age- and PMI-matched controls. Microglial priming is a hallmark of both MIA, associated with increased SCZ risk, and ELA, or the emotional and physical abuse prior to the age of 18 (Tay et al., 2017; Smith and Pollak, 2020). Accordingly, it is tempting to speculate that microglial priming during prenatal and postnatal development contributes to the key role of ELA in SB risk (Figure 1), particularly via impairing neuronal function (Tay et al., 2017), as we discuss next.

#### Prenatal Priming Possibly Disrupts Myelination and Increases Suicidal Behaviors Risk

MIA induced using a maternal high-fat diet results in exacerbated responses of plasma cytokines, such as IL6, to LPS, and decreased microglial interactions with the extracellular space in the HIP of male and female adolescent mouse offspring (Bordeleau et al., 2020), which might be relevant for structural remodeling of synapses or myelination (Nguyen et al., 2020). Indeed, in a follow up study, maternal high-fat diet was associated with decreased postnatal presence of structures that support the axon-myelin unit known as cytosolic myelin channels, in the *corpus callosum* of adolescent males, contrasted by increased microglial synaptic contacts in both sexes (Bordeleau et al., 2021). An altered microglial support of myelination could be connected to the myelin impairment detected in the ACC epigenome and the transcriptome of post-mortem brain samples from individuals with MDD and a history of ELA who died by suicide (Lutz et al., 2017). Considering the marked sexual dimorphism observed in ELA and MIA, it will be crucial that studies addressing this question consider sex differences in microglial behavior.

#### Postnatal Priming Could Increase Microglial Synaptic Pruning Connected to Suicidal Behaviors

Male mice receiving an intraperitoneal injection of LPS during postnatal development display microglial upregulation of CX3CR1 which, in a TLR4-dependent manner, causes heightened synaptic engulfment in the ACC and results in MDD-like symptoms upon acute exposure to unpredictable stressors during adolescence (Cao et al., 2021). Notably, elevated TLR4 and TLR3 expression was found in the post-mortem DLPFC of individuals who died by suicide, irrespective of MDD diagnoses (Pandey et al., 2014). Moreover, the TLR2 was among the top ten genes involved in the DLPFC of individuals with MDD who died by suicide, compared to individuals with MDD who died of other causes and healthy controls (Zeng et al., 2020; Supplementary Table 1). According to the results obtained by Cao et al. (2021), TLR expression changes in post-mortem brain tissue could be indicative of increased postnatal peripheral inflammation leading to microglial synaptic pruning and elevated susceptibility to SB, warranting further investigation.

#### Postnatal Priming Could Impact Future Hypothalamus–Pituitary–Adrenal Function

Postnatal exposure to alcohol in adult male rats increased HYP protein expression of CD11b, a component of complement receptor 3 mediating microglial pruning of axon terminals (Schafer et al., 2012; Chastain et al., 2019). Upregulation of CD11b was accompanied by heightened mRNA levels of *Tnf*, *Il6*, *Tl4*, and *Csf1r* both *in vivo* and *in vitro*, as well as protein levels of epigenetic modulators in microglia from the HYP paraventricular nucleus (Chastain et al., 2019). Particularly, Chastain et al. (2019) revealed a decreased global DNA methylation but increased acetylation of factors that upregulate inflammatory gene transcription, such as histone H3 lysine 9, which prevailed throughout adulthood. Less transcriptional repression in microglia was paralleled by an elevated GC response to a LPS challenge during adulthood and reversed by minocycline (Chastain et al., 2019), a tetracycline normalizer of microglial cytokine release and phagocytosis (Šimončičová et al., 2022). GC are produced by the HPA axis following exposure to physiological or biological threats and have numerous functions, largely affecting inflammatory and metabolic processes (de Kloet et al., 2005; Lucassen et al., 2014; Picard et al., 2021), as well as synaptic plasticity, behavior, and memory (McEwen and Magarinos, 2001). Polymorphisms and differential epigenetic regulation of numerous factors along the HPA axis can underlie impaired stress responses, together with increased psychiatric risk, SB and suicide ideation (Roy et al., 2017; Ke et al., 2018; Menke et al., 2018; Berardelli et al., 2020; Nold et al., 2021). It is possible that inflammatory and epigenetic responses mediated by HYP primed microglia and impacting the HPA support the corresponding increase of SB risk in adolescents exposed to alcohol during development (O’Connor et al., 2019), although warranting more substantial evidence. Additionally, this HPA effect could be mediated via CD11b-complement 3 synaptic remodeling. Correspondingly, decreased mRNA *Cd11b* expression was detected in the ACC of individuals with MDD who died by natural causes but not suicide, compared to age and PMI-matched controls (Zhang L. et al., 2021). Moreover, increased complement protein 3 was detected by microarray in the AMY, HIP, TLM of individuals who died by suicide compared to healthy controls (Glavan et al., 2021). Future studies are needed to explore the role of complement synaptic pruning in SB.

#### Additional Microglial Morphological Analyses Are Required to Inform Suicidal Behaviors Risk

The increased density of primed cells observed by Torres-Platas et al. (2014b) constitutes the only available microglial quantitative morphological analysis in the context of SB. Microglia use cues from the microenvironment to tune their branching processes, as well as the shape and size of their soma, and modulate how motile, mobile and interactive they are with their surroundings (Savage et al., 2019). Enlarged microglial soma size, present in primed cells, was linked to enhanced gene transcription in adult mice (Dungrawala et al., 2010; Kozlowski and Weimer, 2012; Marguerat and Bähler, 2012) and to microglial mobility in aged male and female mice (Hefendehl et al., 2014). Moreover, elevations in IBA1-positive cell area, also detected after priming, were associated with the uptake of neuronal inclusions which coincided with a decreased spine density in the PFC of adult male mice exposed to CUS (Milior et al., 2016; Wohleb et al., 2018). Microglial morphological adaptations related to phagocytosis of neuronal elements were also linked to MDD-like behaviors in CUS-exposed male mice (Milior et al., 2016; Wohleb et al., 2018). Future research is needed to evaluate whether priming-driven changes in microglial morphology are tied to synaptic remodeling and if they could help understand, for example, the impaired reward and decision making, as well as mental pain associated with SB in the DLS and NA (Schmaal et al., 2020). Although microglial morphology is intimately associated to their activity, analyses of microglial morphological states can be highly contradictory, as shown in the stress literature, probably due to experimental variability in the time course and types of stress paradigms used, as well as regions examined and various coping strategies employed (Picard et al., 2021). Qualitative data has argued for a lack of difference in morphology of HLA-DR-positive cells in the DRN of individuals with MDD who died by suicide compared to individuals with MDD who died by other causes and healthy controls (Brisch et al., 2017). In particular, this study compared cells with enlarged cell body and few ramifications, also known as ameboid, and microglia with numerous radial, thin ramifications, defined as ramified (Brisch et al., 2017). Similar lack of SB-specific effects was suggested after comparing the density of HLA-DR ameboid and ramified microglia within the ACC, DLPFC, HIP, and MTN of two individuals with SCZ who died by suicide compared to age-, sex-matched healthy controls (Steiner et al., 2006). Other qualitative assessments, for example, in the ACC and VLPFC, resulted in similar findings (Steiner et al., 2011; Schnieder et al., 2014; Clark et al., 2016; Petrasch-Parwez et al., 2020). Quantitative assessments of microglial morphology are notably required to verify more nuanced differences along the spectrum of morphologies microglia can assume, which goes beyond ramified and ameboid states (Savage et al., 2019). Prospective studies should consider the variety of factors that can influence the shape and size of microglia and contextualize in space and time their morphological and stereological investigations.

### Region-Specific Microglial Heterogeneity May Influence Suicidal Behaviors Risk After Inflammation

Differences of microglial distribution across the CNS are reported in homeostatic contexts, for instance, with higher numbers in cortical regions and lower values in the cerebellum of adult male and female mice (Verdonk et al., 2016; Stowell et al., 2018). Regional microglial heterogeneity is similarly relevant after stress and inflammation (Tan et al., 2020). Upon CRS, only 9 out of 15 CNS regions, including the NA and HIP CA3, presented elevated densities of IBA1-positive cells in adult male rats (Tynan et al., 2010). Similarly, adult male mice showed increased IBA1-positive cell density in the DG 2 days after CUS, while at 5 weeks, a reduction in IBA1-positive cells was observed (Kreisel et al., 2014). In another work, acute but not CSDS resulted in marked increases in CX3CR1-positive cells. Elevated CX3CR1 density was identified in 7 out of 12 regions examined, including the ACC, NA, dorsal DG of adult male mice (Lehmann et al., 2016). Minocycline administration supports that, at least partially, modulation of the inflammatory activity of microglia can rescue changes in density among stress-sensitive areas. In adult male mice receiving IFNα therapy, minocycline rescued increases in density of HIP IBA1-positive cells and mRNA expression of cytokines such as *Ifnα*, *Il6*, *Il1β*, and *Tnf* (Zheng et al., 2015). In the same animals, it similarly restored decreased immunolabeling for the proliferation markers Ki67 and BrdU, neuronal progenitor marker TBR2 and maturation marker DCX, as well as increased MDD-like behaviors (Zheng et al., 2015). Moreover, this antibiotic rescued the effects of CRS in adult male rats, improving spatial working memory and reducing the PFC immunoreactivity for IBA1 and FOSB, which progressively accumulated in repeatedly activated neurons (Hinwood et al., 2012) and is associated with MDD and suicide risk, according to transcriptomic data (Zeng et al., 2020). Minocycline also attenuated increases in IBA1 and C1q protein expression correlated with decreased DRN serotoninergic signaling caused by social isolation-induced alcohol intake in adult male mice (Lee et al., 2021). In adult male rats exposed to ESI, treatment with minocycline further reverted MDD-like behavior, HIP protein IBA1 increases, as well glutamate receptor subunits reductions (Wang et al., 2017). Overall, these results suggest that, in stress-susceptible regions, inflammation potentially affects the density of microglia and impairs synaptic remodeling, which could be relevant to density changes observed upon SB (Figure 1).

#### Region-Specific Elevations in Microglial Numbers Are Detected Upon Suicidal Behaviors

Alike stress, specific CNS regions were more susceptible to changes in IBA1 or HLA-DR upregulation in post-mortem samples. Increased density of cells immunostained for IBA1 or HLA-DR was found in the dACC of male and female individuals with MDD or SCZ who died by suicide (Supplementary Table 1) compared to sex- (Steiner et al., 2006, 2008) and age-matched healthy controls (Torres-Platas et al., 2014b). Elevated HLA-DR was also observed in the DLPFC and MTN of female and male individuals who died by suicide, regardless of their psychiatric diagnosis (Steiner et al., 2006, 2008). Conversely, decreased HLA-DR immunostaining was detected in the DRN of individuals with MDD who did not die by suicide, compared to those who did and to healthy controls (Brisch et al., 2017). By contrast, no significant differences in HIP HLA-DR staining were, however, detected in two individuals with SCZ who died by suicide, compared to healthy matched controls (Gos et al., 2014). Moreover, IBA1-positive cell density in the aMCC did not differ between individuals with SCZ who died by suicide compared to age-matched individuals with SCZ who died by other causes or healthy controls (Petrasch-Parwez et al., 2020).

##### HLA-DR and *IBA1* Signaling Outcomes Are Largely Unknown in the Central Nervous System

As previously discussed, HLA-DR expression is associated with immune signaling, however, studies have yet to explore the possible outcomes of the heterogenous HLA-DR upregulation found in SB. Similarly, while widely used to assess microglial reactivity (Ohsawa et al., 2000), the function of the actin-related protein IBA1 marker is still largely elusive (Walker and Lue, 2015). Depletion of IBA1 can impair HIP microglial motility, leading to deficits in synapse engulfment and increased excitatory synapse number in juvenile mice that impact behavior into adulthood (Lituma et al., 2021). The paradoxical reduction in synaptic uptake and number of excitatory synapses requires further investigation but could indicate that IBA1 signaling participates first in synaptogenesis and later in synaptic pruning during postnatal CNS development (Lituma et al., 2021). Accordingly, it is possible that changes in IBA1 expression detected in post-mortem samples from individuals who died by suicide reflect alterations of synaptic plasticity, rather than inflammation *per se*. Various cognitive alterations are observed in the areas where disturbed post-mortem levels of microglial cells were found, such as the ACC, DLPFC, and MTN. In one study, individuals with MDD and SB displayed a reduced reward anticipation and an increased activation of the ACC after viewing angry faces, as detected by fMRI (Pan et al., 2013; Schmaal et al., 2020). Moreover, a negative correlation between the severity of suicide ideation and resting state fMRI among the MTN was found in adults with MDD (Kim et al., 2017), while deficits in PFC inhibition and flexibility are thought to contribute to the transition between suicide ideation and SB (Schmaal et al., 2020). Possible causal adaptive and maladaptive relationships between the microglial region-specific cell numbers, IBA1, and HLA-DR signaling adaptations (Figure 1) warrant future investigation.

#### Region-Specific Increased Fractalkine Signaling Is Possibly Associated With Suicidal Behaviors Risk

*Cx3cr1* transcripts are increased in the ACC but not in the DLPFC of individuals with SCZ who died by suicide compared to individuals with SCZ who died by other causes (Zhang L. et al., 2021; Supplementary Table 1). Further, CD11b-positive cells from MFG, STG, TLM, and SZ tissue presented increased mRNA expression of *Cx3cr1* when isolated from individuals with MDD compared to age-matched healthy controls (Snijders et al., 2020). According to a microarray study, increased *Cx3cr1* was also detected in the AMY, HIP, and TLM of individuals who died by suicide compared to healthy controls (Glavan et al., 2021). By contrast, no difference in *Cx3cr1* mRNA expression was detected in the ACC or DLPFC of individuals with MDD who died by natural causes and suicide, compared to age and PMI-matched controls (Zhang L. et al., 2021). Microglia and neuronal cells use a wide variety of signaling molecules, including fractalkine, to maintain their balanced communication in the CNS, required for optimal function of both cellular types (Paolicelli et al., 2014). Fractalkine signaling relies on binding of CX3CR1, largely expressed by microglia in the CNS, to the neuronal chemokine fractalkine, or CX3CL1, which generally inhibits microglial activity (Harrison et al., 1998; Biber et al., 2007). CX3CR1-CX3CL1 activity is needed for neuronal maturation and survival, plasticity of synapses and behavior (Paolicelli et al., 2014). During postnatal development, CX3CR1 is required for microglial recruitment to the cortex and maturation of postsynaptic glutamate receptors in female and male mice (Hoshiko et al., 2012). Furthermore, CX3CR1 deficiency impairs HIP neuronal long-term potentiation, a common paradigm to study synaptic plasticity, and results in cognitive and neurogenesis deficits in adult male mice (Bachstetter et al., 2011; Rogers et al., 2011). Given that fractalkine signaling is needed for optimal function of both cellular types (Paolicelli et al., 2014), regional mechanisms involving CX3CR1 could participate in susceptibility to stressors and inflammation contributing to SB risk. Despite the robust role of fractalkine signaling in synaptic plasticity, following CSDS in adulthood, CX3CR1 knockout male mice did not develop anxiety-like behavior compared to wild-type mice, an effect attributed to their lack of peripheral immune cell recruitment to the CNS (Wohleb et al., 2013). Moreover, adult CX3CR1 knockout male mice exposed to CUS failed to modify their HIP microglial morphology, phagocytosis, neuronal plasticity and did not show MDD-like behaviors compared to wild-type mice (Milior et al., 2016; Rimmerman et al., 2017). Similar resistance to developing MDD-like behaviors was found in an adult male and female mouse model of chronic behavioral despair (Hellwig et al., 2016) and in adult male mice exposed to ELA and chronic variable stress in adulthood (Winkler et al., 2017). Additionally, in a human induced pluripotent stem cell microglial model, CX3CR1 knockout resulted in heightened phagocytosis, as assessed with a bead assay (Murai et al., 2020). Region-specific elevations in *Cx3cr1* expression found in post-mortem samples could confer an increased stress susceptibility to microglia, disrupting phagocytosis and neuronal plasticity and contributing to SB (Figure 1), which warrants further research.

## Risk of Suicidal Behaviors May Involve Microglial Oxidative Stress

Oxidative stress is a hallmark of sustained and often maladaptive inflammation (Chaudhari et al., 2014). It refers to the unbalanced production of free radicals, including ROS, which possess unpaired electrons that readily oxidize and modify lipids, DNA and proteins (Bakunina et al., 2015; Black et al., 2015). Despite having homeostatic roles in neurogenesis, synaptic plasticity, programmed cell death and pathogen removal, oxidative stress can severely damage CNS cells. The CNS consumes around 20% of the total oxygen levels in the body but has limited anti-oxidant capacity, therefore, it requires ROS levels to be tightly controlled (Black et al., 2015). As most phagocytic cells, microglia are robust sources of ROS (Block and Hong, 2007). Selective depletion of microglia using the CSF1R antagonist PLX5622 protected adult male mice from the negative behavioral outcomes of CSDS, an effect that was specifically attributed to excessive microglial ROS production in the PFC and HIP (Lehmann et al., 2019). Furthermore, compared to resistant and healthy mice, microglia isolated from the HIP and medial PFC of adult male mice susceptible to CSDS presented higher *ex vivo* labeling with a membrane-permeable dye indicating respiratory burst activity and ROS production (Lehmann et al., 2018). Excessive microglial oxidative stress activity could increase susceptibility to psychiatric and SB risk (Figure 1). Notably, ROS production arises from multiple mechanisms, which could be, simultaneously or not, at play in SB, as we outline next.

### Molecular Evidence of Microglial Oxidative Stress Is Detected Upon Suicidal Behaviors

Microglial ROS can be produced through the activity of NOX2 in response to pathogens and inflammatory molecules (Block and Hong, 2007). Given the inflammatory profile identified in many individuals with SB, microglial NOX2 signaling could be recruited in suicide. In post-mortem brain samples of individuals who died by asphyxiation suicide, NOX2 protein expression was markedly upregulated in cortical GABAergic inhibitory neurons and to a lesser extent in MAC387-positive cells, representing microglia and macrophages, compared to healthy controls and non-suicide asphyxiation deaths (Schiavone et al., 2016; Supplementary Table 1). NOX2 upregulation was accompanied by elevations in IL6 and 8-OHdG, a by-product of ROS-oxidized guanine (Schiavone et al., 2016). Elevated 8-OHdG levels were similarly detected in the CA1, CA2 and DG of male and female individuals with MDD or SCZ compared to controls (Che et al., 2010). While looking at death by asphyxiation, the work by Schiavone et al. (2016) highlights that specific methods of suicide are likely associated with different impacts on oxidative or inflammatory pathways affecting microglial function, an area that awaits future investigation.

### Mitochondrial Oxidative Stress Is Associated With Suicidal Behaviors

ROS can similarly originate from the activity of the mitochondrial respiratory chain (Wilkinson and Landreth, 2006). Twenty subunits of the mitochondrial oxidative phosphorylation complexes showed increased levels in the DLPFC of individuals with MDD, whilst being usually decreased in SCZ (Martins-de-Souza et al., 2012). Furthermore, a suicide-specific elevation in the expression of DNA-dependent ATPase activity was revealed by gene ontology analysis in the DLPFC, AMY, and TLM (Pantazatos et al., 2017; Supplementary Table 1). Higher oxidative phosphorylation activity was hypothesized to compensate for oxidative stress which depletes ATP production in the DLPFC of individuals with MDD (Martins-de-Souza et al., 2012). Despite looking at post-mortem tissue from suicide deaths, the study by Martins-de-Souza et al. (2012) did not discuss the SB-specific proteomic changes in their cohort, encouraging further research in this topic. Additionally, after CUMS, adult male mice exhibited altered mitochondrial ultrastructure, such as swelling, disrupted cristae and membranes and impaired respiration rates among the cortex, HIP, and HYP, paralleling MDD-like behaviors (Gong et al., 2011). Overall, these results indicate mitochondrial respiratory chain oxidative stress is seen upon SB and stress.

#### Dark Microglia May Participate in Suicidal Behaviors After Oxidative Stress and Epigenetic Modulation

Disrupted mitochondria, dilation of the Golgi apparatus and endoplasmic reticulum, as well as cell shrinkage and abundant endosomes, are hallmarks of DM, a microglial state uncovered by our group (Bisht et al., 2016). DM often encircle synaptic structures and are occasionally surrounded by extracellular space containing debris, altogether suggesting ongoing synaptic pruning and extracellular digestion (Bisht et al., 2016). Albeit nearly absent during mature steady-state conditions in the HIP, PFC, HYP, and BLA, DM become abundant in male mice subjected to MIA and in mouse models of stress, including CSDS and CUMS (Bisht et al., 2016). Given the appearance of DM upon distal and proximal SB risk factors as well as their marked oxidative stress, it is tempting to speculate that DM are potentially found upon SB. Electron microscopy studies assessing post-mortem samples from suicide deaths could clarify this matter. Notably, “dystrophic” microglia that resemble DM are present in the post-mortem PFC of individuals with SCZ (Uranova et al., 2018). Correspondingly, DM could help explain the dendritic atrophy and reduced spine density found in the PFC and HIP of male and female rats and mice exposed to CRS and CUMS (Qiao et al., 2016), similar to what is observed in the same regions of individuals with MDD (Duman et al., 2016; Bollinger and Wohleb, 2019).

In addition to marked oxidative stress, DM are characterized by an electron-dense nucleus with altered nuclear heterochromatin (Bisht et al., 2016; St-Pierre et al., 2020). Nuclear heterochromatin remodeling is a process coupled to DNA methylation and transcriptome adaptations (Robertson, 2002). Notably, DNA methylation is one of the proponent mechanisms by which ELA translates into higher SB risk later in life (Lutz et al., 2017; Zeng et al., 2020). ELA-induced methylation could potentially drive the appearance of DM, albeit more direct evidence is needed to support this hypothesis. Initial studies indicate microglia can be the target of complex epigenetic modulation after exposure to psychological stress, such as ESI and ESS (Wang et al., 2017; Catale et al., 2020), or physiological challenges, for instance early-life alcohol exposure (Chastain et al., 2019). IBA1-positive cells located in the core and shell of the NA, dorsomedial striatum and DLS, CA1 and CA3 of the HIP, as well as BLA and central AMY showed a marked decrease of methylation in ESI mice compared to control and ESS groups (Catale et al., 2020). Additionally, the authors revealed a decreased global methylation in the CA1 of mice exposed to ESS compared to controls from both sexes (Catale et al., 2020). While similar mechanisms might participate in SB, microglial epigenetic studies in the context of SB neuropathology are currently lacking.

### Impaired Tryptophan Metabolism Induced by Oxidative Stress May Affect Suicidal Behaviors Risk

Cytokines and oxidative stress molecules work in a positive feedback loop (Black et al., 2015; Figure 1). ROS stimulation results in activation of NF-κB and production of cytokines (Bakunina et al., 2015; Black et al., 2015). In turn, increased protein levels of IFNγ, IL6, IL1β, and TNF enhance oxidative stress (Bakunina et al., 2015; Black et al., 2015). Cytokines and ROS, both notably released by microglia, similarly activate IDO and TDO, which metabolize L-tryptophan through the kynurenine pathway (Heyes et al., 1992, 1996; Alberati-Giani et al., 1996; Fujigaki et al., 2017). In humans, IDO is expressed by microglia (Figure 1), astrocytes and to a lesser extent in neurons, whilst TDO is mostly detected in astrocytes (Wu et al., 2013). Decreased kynurenine:tryptophan ratio was identified in the VLPFC of individuals with MDD compared to healthy controls, irrespective of the cause of death, including suicide (Clark et al., 2016; Supplementary Table 1). In agreement, this study detected lower mRNA expression of *Ido1* and *Ido2*, their homologous genes, as well as *Tdo*, in the VLPFC of individuals with MDD (Clark et al., 2016). In adult male rats, following LPS injection, mRNA upregulation of *Ido* was found to be accompanied by increased transcript levels of *Tnf* and *Il6* in the cortex and HIP (O’Connor et al., 2009). While it is unclear if a similar upregulation is present in SB, a polymorphism in the promoter region of the IDO1 gene is associated with higher risk of developing MDD symptoms in individuals receiving IFN therapies (Smith et al., 2012), suggesting that IDO1 plays a significant role in determining the susceptibility to SB.

#### Microglial Tryptophan Metabolites Are Regionally Detected in Suicidal Behaviors

In the brain, L-kynurenine gets broken down into 3-hydroxykynurenine, QUIN and xanthurenic acid, all synthesized by microglia and peripheral macrophages according to human cells *in vitro* evidence (Heyes et al., 1992, 1996; Espey et al., 1997; Gos et al., 2014), while kynurenic acid and picolinic acid are produced by astrocytes (Raison et al., 2006; Fujigaki et al., 2017). QUIN is a marker of oxidative stress which was found to be significantly elevated in the CSF of individuals with a history of suicide attempts (Erhardt et al., 2013) and in the ACC of individuals with MDD who died by suicide compared to age- and sex-matched healthy controls (Steiner et al., 2011; Supplementary Table 1). In the same cohort, however, decreased QUIN immunoreactivity was found in the HIP CA1 of individuals with MDD or bipolar disorder who died by suicide compared to age- and sex-matched healthy controls, indicating that different brain regions likely present distinct microglial oxidative balances in SB (Busse et al., 2015). Similarly, individuals with SCZ presented reduced QUIN binding in the CA1 compared to controls without psychiatric diagnoses, despite a lack of specific changes in the two suicide deaths included in this study (Gos et al., 2014). Robust evidence links oxidative stress and SB; however, more studies are warranted to explore the extent through which microglia contribute to modulating region-specific tryptophan metabolism in particular mental health disorders and SB (Suzuki et al., 2019; Baharikhoob and Kolla, 2020).

#### Impaired Tryptophan Metabolism Disrupts Serotonin Levels and Microglial Function

Tryptophan is a precursor of serotonin (Bakunina et al., 2015; Fujigaki et al., 2017). Increased microglial degradation of tryptophan, supported by changes in IDO isoforms and QUIN levels, could help explain the decreased levels of serotonin metabolites measured in the CSF of individuals with MDD and SB (Brown et al., 1982; Mann et al., 1992; Steiner et al., 2011; Bakunina et al., 2015; Fujigaki et al., 2017; Figure 1). Notably, serotonin depletion is associated with increased impulsivity and aggressive behaviors (Stanley et al., 1982; Stanley and Mann, 1983), low mood, anxiety, as well as suicide risk (Achtyes et al., 2020; Baharikhoob and Kolla, 2020; Koweszko et al., 2020). More research could clarify the specific contribution of microglia to serotonin depletion in the context of SB. Simultaneously, serotonin affects the inflammatory activity, trophic support, phagocytosis and motility of microglia in a context-dependent manner (Turkin et al., 2021). For instance, serotonin treatment enhanced adult mouse cortical microglial process motility toward laser injury but reduced phagocytosis of beads by neonatal amoeboid microglia of the *corpus callosum in situ* (Krabbe et al., 2012). Adult male mice treated with the selective serotonin reuptake inhibitors fluoxetine exhibited different HIP inflammatory responses according to the quality of their living environment (Alboni et al., 2016). In mice exposed to CUMS, fluoxetine was associated with anti-inflammatory responses, such as reduced *Tnf* transcripts, increased microglial spacing index and cell body area, but decreased arborization in the HIP CA1 (Alboni et al., 2016). Conversely, in mice housed in an enriched environment, microglia had increased pro-inflammatory responses, involving IL1β protein and *Tlr4* mRNA (Alboni et al., 2016). Stress appears to regulate serotonin levels along with microglial activity. Social isolation in adult male mice caused a decrease in DRN serotonin production accompanied by elevated IBA1 and C1q protein expression, as well as depressive-like behavior (Lee et al., 2021). Upregulation in complement expression was paralleled by a downregulation in synaptic proteins, i.e., postsynaptic density protein 95 and synaptophysin, which could indicate synaptic pruning (Lee et al., 2021). It is therefore possible that stress-induced serotonin depletion increases the susceptibility to psychiatric disorders or SB via adaptations in microglial pro-inflammatory activity, cell motility and microglial synaptic remodeling, an area that awaits future investigation.

#### Microglial Tryptophan Metabolites Can Cause Glutamate Excitotoxicity

In LPS-treated adult male mice, minocycline and the IDO antagonist 1-methyltryptophan rescued the development of MDD-like behavior, independently of brain serotonin turnover (O’Connor et al., 2009). Therefore, at least partially, microglial IDO may influence SB independently of serotonin. As previously described, in addition to reducing tryptophan levels, IDO activity results in production of the metabolite QUIN in microglia. QUIN is an agonist of the NMDAR crucial for glutamatergic excitatory signaling in the CNS (Olloquequi et al., 2018). Elevated levels of QUIN, found in association with SB, as discussed above (see section “Microglial Tryptophan Metabolites Are Regionally Detected in Suicidal Behaviors”), can over-activate the NMDAR, generating abnormally high levels of intracellular Ca^2+^, toxic free radicals and disrupted ATP production that ultimately contribute to loss of neuronal cell function and cell death, a process formally known as glutamate excitotoxicity (Olloquequi et al., 2018). QUIN-mediated glutamate excitotoxicity could be another mechanism by which microglia participate in SB (Suzuki et al., 2019; Baharikhoob and Kolla, 2020; Figure 1). Correspondingly, several clinical trials with ketamine, a NMDAR antagonist, found a significant and rapid reduction in suicidal cognition, a reason why ketamine therapy is currently being evaluated for the treatment of suicidal ideation (Ballard et al., 2021). Ketamine was shown to rescue MDD-like behavior, decreasing brain IL6, TNF and QUIN protein production, as well as CX3CR1-positive cell area, whilst increasing CX3CR1-positive cell arborization in the PFC of adult male mice exposed to LPS (Verdonk et al., 2019). Partial depletion of microglia by the CSF1R inhibitor PLX3397 blocked the effects of R-ketamine, a more potent and longer-lasting enantiomer of (R,S)-ketamine in adult male mice susceptible to CSDS (Zhang K. et al., 2020). Hence, ketamine affects microglia in a manner that is partially required to achieve its anti-depressant effects. It is, however, unknown if similar mechanisms underlie the effects of ketamine in SB.

Despite positive results in the context of MDD, an inverse response to ketamine is found in SCZ, where it produces psychosis-like responses in healthy subjects and can temporarily worsen positive symptoms in individuals previously diagnosed with SCZ (Lahti et al., 2001). This indicates that NMDAR antagonism is to some degree involved in SCZ pathology (Stone et al., 2007; Straub et al., 2007; Nakazawa et al., 2012; Steiner et al., 2012) and that ketamine may possibly induce disorder-specific adaptations in microglia. It is postulated that hypofunctional NMDAR located on GABAergic inhibitory interneurons disinhibit excitatory pyramidal neurons, the principal neurons of the cerebral cortex, leading to a paradoxical increase in glutamatergic activity that contributes to SCZ (Stone et al., 2007; Nakazawa et al., 2012). Based on cortical post-mortem results of asphyxiation suicide deaths, cytokines such as IL6 affecting NOX2 expression in GABAergic neurons, can contribute to oxidative stress that causes the loss of inhibitory tone on glutamatergic neurons and increases glutamatergic excitotoxicity (Schiavone et al., 2016; Supplementary Table 1). Cortical glutamate excitotoxicity is hypothesized to elevate synaptic apoptosis, a sub-lethal form of apoptosis in terminal neurites and individual synapses that triggers synaptic elimination in the absence of cell death, in line with the reduced cortical volume in individuals with SCZ without associated pyramidal cell loss (Bennett, 2011; Schobel et al., 2013; Parellada and Gassó, 2021). While in the DLPFC and HIP apoptosis and neuronal death pathways were significantly correlated with suicide death (Zeng et al., 2020; Glavan et al., 2021), further studies are required to evaluate whether these changes reflect cellular or synaptic events. It is suggested that synaptic apoptosis causes exposure of internal phosphatidylserine and accumulation of complement on the synaptic membrane, such as C1q, which stimulate microglial chemotaxis and synaptic pruning (Nonaka and Nakanishi, 2019; Parellada and Gassó, 2021). Minocycline successfully dampened the aberrant synaptic engulfment of SCZ patient-derived microglial cells *in vitro* (Sellgren et al., 2019) and may be used for similar outcomes in SB.

##### Elevated Purinergic Signaling May Contribute to Suicidal Behaviors Risk

Glutamate excitotoxicity elevates extracellular levels of ATP in HIP slices of adult female and male mice (Dissing-Olesen et al., 2014), similar to what is observed in male rats exposed to CUS, which can develop MDD behaviors upon chronic ATP administration (Yue et al., 2017). In mice, cortical P2RY12 binds to extracellular nucleotides, like ATP, and is required for postnatal synaptic plasticity (Sipe et al., 2016), in addition to microglial chemotaxis and phagocytosis (Davalos et al., 2005; Haynes et al., 2006; Dissing-Olesen et al., 2014), as well as BBB closure (Lou et al., 2016), upon injury in adult males. Elevated *P2ry12* transcripts are found across the ACC of individuals with SCZ who died by suicide compared to individuals with SCZ who died by other causes (Zhang L. et al., 2020; Supplementary Table 1). Correspondingly, a single-cell microglial study of medicated individuals with MDD has found elevations in P2RY12 protein expression in the frontal lobe, temporal lobe, TLM and SZ, in parallel with a decrease in HLA-DR and CD68 compared to age-, sex- and PMI-matched controls (Böttcher et al., 2020). Moreover, decreased mRNA *P2ry12* expression was detected in the ACC of individuals with MDD who died by natural causes but not suicide, compared to age and PMI-matched controls (Zhang L. et al., 2020). Upregulation of P2RY12 without exacerbated inflammation may indicate increased neuron-microglia communication (Böttcher et al., 2020). It is possible that, after stress, ATP-P2RY12 recruitment attracts microglia toward neurons with an elevated excitatory activity, promoting synaptic pruning to reduce excitability along with psychiatric-like behavior (Bollinger and Wohleb, 2019) and SB. Additional studies are warranted to explore the putative origins and outcomes of purinergic signaling in the context of SB.

##### Balance Triggering Receptor Expressed on Myeloid Cells 2 Signaling May Reduce Suicidal Behaviors Risk

*Trem2* transcripts were reduced in the ACC of individuals with SCZ, but only in deaths not caused by suicide (Zhang L. et al., 2020; Supplementary Table 1). In opposition, mRNA *Trem2* expression was not significantly different in the ACC or DLPFC of individuals with MDD who died by natural causes and suicide, compared to age and PMI-matched controls (Zhang L. et al., 2021). TREM2 is a cell surface protein binding to phospholipids, phosphatidylserine, sulfatides, LPS and DNA, which is involved in amyloid β and apoptotic neuron clearance, as well as AD risk (Guerreiro et al., 2013; Jonsson et al., 2013; Wang et al., 2015; Krasemann et al., 2017). MDD risk genes were involved in the pathology of AD (Ni et al., 2018), but it is unclear whether TREM2 signaling is affected (Santos et al., 2016). Balanced TREM2 levels could be a protective factor against the apoptotic effects of glutamate excitotoxicity (Ren et al., 2021) in SB, although requiring more substantial research. Studies with TREM2 knockout mice suggest this protein can reflect microglial proliferation, survival around amyloid β and migration toward injury throughout life (Ulland and Colonna, 2018). Moreover, TREM2 absence impairs postnatal microglial synaptic pruning, consequently increasing HIP excitatory neuronal activity and stereotypic behavior in adult female and male mice (Filipello et al., 2018). Accordingly, elevated microglial TREM2 protein expression reduced microglial production of the pro-inflammatory cytokines TNF and IL1β, but increased phagocytosis *in vitro* (Takahashi et al., 2005). Additional studies are needed to clarify the outcomes of reduced TREM2 expression in psychiatric or SB risk.

## Microglial Neuronal Trophic Support Could Be Impaired in Suicidal Behaviors

Microglia could additionally influence plasticity in SB by altering the release of neurotrophins, such as BDNF (Dwivedi, 2009). Individuals with a history of suicide attempts and MDD present reduced levels of plasma BDNF compared to control groups (Dawood et al., 2007; Deveci et al., 2007; Kim et al., 2007; Lee et al., 2007), although a larger study found a lack of effect (Eisen et al., 2016). Moreover, decreased BDNF protein expression was detected in the PFC and HIP of individuals who died by suicide, compared to healthy controls (Dwivedi et al., 2003; Banerjee et al., 2013). Similarly, BDNF protein level was lower in the ACC, but not the DLPFC, in suicide decedents with a history ELA compared to controls without reported adversity or suicide death (Youssef et al., 2018). *In vivo* and *in vitro* data from adult male rats indicates microglial BDNF can cause neuronal hyperexcitability in the spinal cord relevant to pain disorders (Coull et al., 2005). In adult mice, abrogation of microglial BDNF is enough to recapitulate the effects of CX3CR1 cells depletion in motor learning tasks and cortical dendritic spine remodeling (Parkhurst et al., 2013). Moreover, susceptibility to CUMS in adult male mice was associated with reduced HIP arginase-positive microglial IL4 signaling and decreased microglial production and secretion of BDNF (Zhang J. et al., 2021). Dysfunctional microglial BDNF production and synaptic remodeling activity as a result of susceptibility to stress could help explain changes in neurotransmitter binding and dendritic spine density observed in individuals with SB (Kang et al., 2012; Underwood et al., 2012, 2020; Dean et al., 2016; Holmes et al., 2019). Altogether, these results emphasize the intricate and multidimensional microglial activities putatively involved in SB, encouraging further studies in this area.

## Considerations on Microglial Diversity

Recent research has uncovered that the widespread and diverse functions of microglia are fulfilled by a spectrum of cellular states with variable morphology, proteome, metabolome and ultrastructure (Stratoulias et al., 2019). Microglial diversity is drawn by cues from their microenvironment, which differ according to the species, age, sex, CNS region, particular context of health or disease, as well as lifestyle habits of the organism (Hanamsagar and Bilbo, 2017; Bordeleau et al., 2019; Masuda et al., 2020). Context-dependent microglial diversity challenges the entangling of suicide-specific microglial mechanisms. As a primary constraint, results from animal models of SB risk factors may not completely translate to humans. For example, despite sharing a generally similar transcriptome with mouse microglia, human microglia isolated from surgical procedures presented a species-specific expression bias for several transcripts, including those associated with complement proteins, crucial for synaptic turnover (Gosselin et al., 2017). Secondly, great variability in the distribution of sex, age, PMI and psychiatric diagnoses is present among samples examined thus far (Supplementary Table 1), straining inter- and intra-studies comparisons. For example, the PMI between studies fluctuates from 7 to 40 h (Supplementary Table 1) and was shown to correlate with some of the gene expression and morphological alterations detected in microglia (Steiner et al., 2006; Gos et al., 2014; Torres-Platas et al., 2014b). Acutely tuned to environmental cues, microglia appear to change from the time of death until tissue fixation or cell isolation. Their dynamic processes still respond to axonal lesions of the spinal cord up to 10 h after the death of adult mice (Dibaj et al., 2010). Yet, post-mortem microglial responses are not expected to last longer than this given the depletion of immediate energy sources such as ATP (Dibaj et al., 2010), oxygen and glucose (Eyo and Dailey, 2012). Moreover, it is predicted that, even in the best scenarios of body storage at 4^°^C, some RNA, DNA and protein degradation occurs, thus, hindering multi-omic approaches and immunohistochemistry assays (Ferrer et al., 2008). Evidence from a mouse and human study, however, suggests that it is still possible to obtain viable microglia and high-quality RNA after 12 h PMI, with relatively few transcriptional changes compared to shorter PMIs (Heng et al., 2021). Correspondingly, one adult male mouse brain harvested and fixed after a PMI of 43 h mostly displayed similar numbers of ramified IBA1-positive cells in the ACC compared to brains immediately harvested after death (Torres-Platas et al., 2014a). Method of death could similarly create acute brain changes related to hypoxia and oxidative stress. To discriminate SB-specific changes, previous post-mortem studies investigating microglia have suggested matching suicide deaths groups with controls who died under stressful or inflammatory conditions, including homicide and death related to cardiovascular disease (Steiner et al., 2008; Clark et al., 2016). In addition to PMI or method of death, inter-individual differences in coping strategies, risk factor exposure, for instance, ELA, as well as factors such as sex, CNS region and age could help explain the heterogenous results concerning microglial gene and protein expression, as well as morphology found across studies in SB (Supplementary Table 1). Notably, significant efforts are already present in the literature to control for confounders using age-, sex-, tissue pH-, PMI-, and psychiatric diagnoses-matched cohorts (Steiner et al., 2006, 2008, 2011; Gos et al., 2014; Torres-Platas et al., 2014a; Busse et al., 2015; Schneider et al., 2015; Pantazatos et al., 2017; Petrasch-Parwez et al., 2020; Snijders et al., 2020; Glavan et al., 2021). It will be crucial that prospective studies continue to strive to contextualize in space and time their analyses of microglia.

## Conclusion

Suicide has an important neurobiological component predisposed by cumulative risk factors throughout life, namely stress and psychiatric disorders. Abundant and diverse evidence posit microglia as mechanistically involved in the neurobiological etiology of SB and its risk factors. In this Review, we summarized various promising microglial pathways deriving from stress-induced inflammation, oxidative stress and trophic support that could be further investigated in the context of SB. Our discussion centered around putative outcomes on neuronal activity but also included BBB function, and other immune populations infiltrating the CNS. Moreover, we outlined how diversity in microglial states, functions and features, influenced by factors such as region, sex, age and lifestyle, is a key component to explore in future SB research. According to current data, it is likely that microglia participate in mechanisms that contribute to both the resistance and susceptibility to SB after stress. Selective modulation of processes that boost resistance to neuropathology encountered in SB could help prevent suicide deaths.

## Author Contributions

EGA, FGI, and M-ÈT conceptualized the manuscript. EGA prepared the tables and figure with input from FGI and M-ÈT. All authors contributed to the writing and editing of the manuscript.

## Conflict of Interest

The authors declare that the research was conducted in the absence of any commercial or financial relationships that could be construed as a potential conflict of interest.

## Publisher’s Note

All claims expressed in this article are solely those of the authors and do not necessarily represent those of their affiliated organizations, or those of the publisher, the editors and the reviewers. Any product that may be evaluated in this article, or claim that may be made by its manufacturer, is not guaranteed or endorsed by the publisher.
