# Supplementary material for: Microglia as a Hub for Suicide Neuropathology: Future Investigation and Prevention Targets
Source: Front Cell Neurosci. 2022 May 18;16:839396. doi: 10.3389/fncel.2022.839396 (PMC9158339; doi:10.3389/fncel.2022.839396)
Supplement: Supplementary file 1 [file Table_1.pdf]

Supplementary Table 1. Studies investigating microglial properties or functions in SB.

Ref.	*Con.*	*Sample*	*Diag.*	*Mean age*	*PMI*	*Brain areas*	*Methodology*	*Main results*	*Brain Bank*
**Steiner, J et al. 2006**	CT	n = 16<br>F = 8<br>M = 8	N/A	58	37	ACC<br>DLPFC<br>HIP<br>MTN	PB formaldehyde and paraffin.<br>HLA-DR IHC.<br>Qualitative analysis of ameboid (enlarged cell body and few, short processes) and ramified (normal appearing soma and thin, radial processes) microglia.<br>Density analysis of ameboid and ramified microglia per hemisphere.<br>Monocytes identified as HLA-DR-positive cells inside vessels.	Elevated HLA-DR-positive cell numbers in the ACC and MTN in SD.<br>Ameboid microglia lateralized towards the right hemisphere in DLPFC and MTN of CT.<br>No particular effect of SD on qualitative microglial morphology.	MBC
	SD	n = 2<br>F = 1<br>M = 1	SCZ	50	24				
**Steiner, J et al. 2008**	CT	n = 10<br>F = 5<br>M = 5	N/A	54	39	ACC<br>DLPFC<br>HIP<br>MTN	Formalin and paraffin.<br>HLA-DR IHC.<br>Qualitative analysis of ameboid (enlarged and oval cell body and few, short processes) and ramified (thin, radial processes) microglia.<br>Monocytes identified as HLA-DR-positive cells inside vessels.	Increased HLA-DR-positive cells density in the DLPFC, ACC, MTN of individuals who died by suicide regardless of diagnosis.<br>HLA-DR was detected in ramified and ameboid microglia.	MBC
	SD	n = 6<br>F = 4<br>M = 2	SCZ	50	41				
	NSD	n = 10<br>F = 5<br>M = 5	SCZ	55	50				
	SD	n = 7<br>F = 4<br>M = 3	MDD or BD	41	38				

Ref.	*Con.*	*Sample*	*Diag.*	*Mean age*	*PMI*	*Brain areas*	*Methodology*	*Main results*	*Brain Bank*
	NSD	n = 7<br>F = 4<br>M = 3	MDD or BD	51	39				
**Steiner, J et al. 2011**	CT	n = 10<br>F = 5<br>M = 5	N/A	56	35	aMCC<br>sACC<br>pACC	Formalin and paraffin.<br>QUIN IHC.<br>Morphological criteria to distinguish microglia, astrocytes, oligodendrocytes or neurons.<br>Monocytes classified as cells in vessels. Only parenchymal cells were included in the analysis.<br>Qualitative analysis of microglial morphology based on smooth or granular processes and ovoid or elongated cell form.	Increased QUIN-positive cell density in the sACC and the aMCC of SD.<br>CT had mostly a smooth, ovoid, or elongated cell form but individuals with MDD had forms with numerous granular structured processes in aMCC and the sACC.<br>No particular effect of SD on qualitative microglial morphology.	MBC
	SD	n = 12<br>F = 6<br>M = 6	MDD or BD	51	35				
**Torres-Platas, S et al. 2014**	CT	n = 17<br>F = 1<br>M = 16	N/A	39	45	dACC	Formalin.<br>IBA1 and CD45 IHC.<br>Semi-stereological quantification of ramified, primed, reactive, and amoeboid microglia defined based on IBA1 staining and morphology.<br>Macrophages assessed based on CD45 immunostaining and apposition to blood vessel.<br>Fresh frozen samples used for mRNA quantification of IBA1, CD68, CD45, MCP1, SE, CateninA1, ZO1, IL1β, IL10, TNF, IL1RA and GAPDH.	Increased density of IBA1-positive primed microglia, of blood vessels associated with IBA1-positive cells and of IBA1, CD45, MCP-1, ZO1 mRNA in SD compared to CT.<br>No differences in IL1β, IL1Ra, TNF, IL10, CD68, SE, Catenin-a1.	DBCBB
	SD	n = 24<br>F = 6<br>M = 18	MDD	46	42				

Ref.	*Con.*	*Sample*	*Diag.*	*Mean age*	*PMI*	*Brain areas*	*Methodology*	*Main results*	*Brain Bank*
**Schnieder, T et al. 2014**	NSD	n = 25<br>F = 12<br>M = 13	AD, SCZ, or N/A	55[a]	15	dPFWM<br>vPFWM	PB formalin and paraffin.<br>IBA1 and CD68 IHC.<br>Macrophage, resting (thin processes, punctate CD68), and activated (thicker processes, abundant CD68) microglia classification based on morphology and CD68 staining.<br>Activated microglia and macrophages were combined to estimate the density of activated phagocytes.<br>Stereological estimation of microglial density.	Increased density of IBA1-positive perivascular cells in DPFWM of SD.<br>No significant differences of SD on the density of resting or activated microglia.<br>Increased density of activated microglia ventrally *versus* dorsally among SD, and the opposite effect for NSD.<br>No particular effect of SD on qualitative microglial morphology.	NYSPI
	SD	n = 11<br>F = 6<br>M = 5	AD, SCZ, or N/A	56[a]	12				
**Busse, M et al. 2014**	CT	n = 10<br>F = 5<br>M = 5	N/A	56[b]	35	CA1<br>CA2/3	PB formaldehyde and paraffin.<br>QUIN IHC.<br>Monocytes classified as cells inside vessels.<br>Only parenchymal cells were included in the analysis.<br>Hippocampal volume.	Decreased QUIN-positive cells in the right CA1 of SD compared to age- and sex-matched CT.<br>No differences in hippocampal volume.	MBC
	SD	n = 12<br>F = 5<br>M = 7	MDD or BD	50[b]	29				
**Gos, T et al. 2014**	CT	n = 12<br>F = 6<br>M = 6	N/A	49	35	CA1<br>CA2/3<br>DG	PB formaldehyde and Paraplast®.<br>QUIN and HLA-DR IHC.<br>Monocytes classified as cells inside vessels.<br>Only parenchymal cells were included in the analysis.<br>Qualitative assessment of ameboid and ramified microglia based on unclear criteria.<br>Hippocampal volume.	No significant difference in QUIN-positive or HLA-DR-positive cells in SD compared to individuals with SCZ who did not die by suicide or CT.<br>Ameboid and ramified cells not counted separately.	MBC
	SD	n = 2<br>F = 1<br>M = 1	SCZ	51	39				

Ref.	*Con.*	*Sample*	*Diag.*	*Mean age*	*PMI*	*Brain areas*	*Methodology*	*Main results*	*Brain Bank*
**Schiavone, S et al. 2016**	CT	n = 10<br>F = 2<br>M = 8	N/A	[d]See legend	24 - 48	Cortex	Paraffin.<br>HE staining.<br>NOX2, 8-OHdG, IL6, TNF, IL10, IL1β, NeuN, GFAP, MAC387, GAD67, VGLUT1, DT1 IHC.	Increased NOX2-, 8-OHdG-, or IL6-positive cell density in ASD group compared to NSAD and CT.<br>Most NOX2 expressed in NeuN-positive cells and less in MAC387-positive cells.<br>No significant differences for TNF, IL10, IL1β.	SLM
	NSAD	n = 6<br>F = 4<br>M = 2	N/A						
	ASD	n = 26<br>F = 5<br>M = 21	[c]See legend						
**Clark, S et al. 2016**	CT	n = 36<br>F = 9<br>M = 27	N/A	42	30	VLPFC	Frozen tissue.<br>IBA1 IHC.<br>Qualitative morphology analysis of hypertrophic microglia (long, thick hyper-ramified processes).<br>RT-qPCR of IDO, IDO2, TDO2, TNF, IFNγ, IL13, IL33, IL2, CCL2, IL1β, IL6, IL4, IL5, COX2.<br>KYNA and 3-HK High-performance liquid chromatography.<br>TRYP, KRNA and QUIN gas chromatography/mass spectrometry.<br>Nissl, HE, acidic toluidine blue staining.	Decreased KYNA:TRYP ratio and lower mRNA expression of the IDO1, IDO2 and TDO2 in MDD group, irrespective of the cause of death, including suicide.<br>Higher values in CT for TNF, IFNγ, IL13, IL33, IL2 and CCL2. No differences for IL1β, IL6, IL4, IL5, COX2.<br>No differences between groups for intensely stained hypertrophic microglia, characterized by long, thick hyper-ramified processes.	CBDB
	SD	n = 25[e]	MDD	[e]See legend	[e]See legend				
**Brisch, R et al. 2017**	CT	n = 22<br>F = 15<br>M = 7	N/A	52	24	DRN	PB formaldehyde and paraffin.<br>HLA-DR IHC.<br>Argyrophilic nucleolar organizing region silver, Nissl, myelin staining.	Decreased HLA-DR-positive cell density in individuals with NSD compared to SD and CT.	MBC

Ref.	*Con.*	*Sample*	*Diag.*	*Mean age*	*PMI*	*Brain areas*	*Methodology*	*Main results*	*Brain Bank*
	SD	n = 24<br>F = 13<br>M = 11	MDD, BD, or SCZ	48	24		Ramified (thin, radial processes) and ameboid (enlarged ovoid cell body and few short processes) microglia defined based on morphology and HLA-DR staining. Only cells located in the parenchyma were evaluated.	No differences in morphology between SD and NSD cohorts.	
	NSD	n= 21<br>F = 10<br>M = 11	MDD, BD, or SCZ	54	48				
**Pantazatos, S et al. 2017**	CT	n = 29<br>F = 6<br>M = 23	N/A	44	13	DLPFC	Frozen tissue.<br>Whole-exome gene and exon expression analysis.<br>PCR validation.<br>GO analysis.	Lower expression of pathways involved in immune-related and microglial cellular functions (i.e. cellular response to type 1 interferon, regulation of leukocyte chemotaxis, regulation of cytokine biosynthetic process), of regulation of synaptic transmission, glutamatergic in NSD.<br>Higher expression of pathways involved in DNA-dependent ATPase activity' in SD.<br>Most significant GO gene sets include chemokine receptor binding and cellular response to LPS, all lower in individuals with MDD, with suggestive evidence for lowest expression in the SD.<br>No significant differences in	DMIN
	SD	n = 21<br>F = 8<br>M = 13	MDD	52	16				
	NSD	n = 9<br>F = 3<br>M = 6	MDD	58	15				
	CT	n = 9<br>F = 3<br>M = 6	N/A	57	17		Frozen tissue<br>microRNA analysis.		
	SD	n = 9<br>F = 3<br>M = 6	MDD	59	13				

Ref.	*Con.*	*Sample*	*Diag.*	*Mean age*	*PMI*	*Brain areas*	*Methodology*	*Main results*	*Brain Bank*
	NSD	n = 9<br>F = 3<br>M = 6	MDD	58	15			miRNAs or gene variants.<br>PCR results were, in general, consistent with RNAseq data.	
**García Gutierrez, M et al. 2018**	CT	n = 15[f]	N/A	46	15	DLPFC	Frozen tissue.<br>RT-PCR and Western blot of CB2 and GPR55.<br>*In situ* proximity ligation assays.<br>IFL GFAP and IBA1.	Lower levels of CB2rA, GPR55 mRNA in the DLPFC of SD compared to CT.<br>Higher CB2-GPR55 heteroreceptor complexes per cell in the SD group, present in astrocytes and neurons but not microglia.	ILM
	SD	n = 18<br>M = 18	N/A	43	17				
**Holmes, et al. 2018**	CT	n = 13<br>F = 6<br>M = 7	N/A	33	N/A	ACC<br>PFC<br>Insula	Prototypical TSPO radioligand [11C](R)-PK11195 to measure brain TSPO availability through PET scan.<br>Body mass index, childhood adversity and physical exercise measured through questionnaires.<br>Plasma TNF, IFNγ,<br>IL6, IL8, IL1β and CRP.	Higher TSPO availability across ACC, PFC, and insula in individuals with MDD compared to CT.<br>Higher TSPO availability in ACC and insula in individuals with ST compared to CT and NST.<br>No difference in concentration of peripheral inflammatory markers between ST and NST.<br>No relationship between TSPO binding and BMI, childhood adversity or physical exercise.	N/A
	ST	n = 9[g]	MDD	[g]See legend	N/A				
	NST	n = 5[g]	MDD	[g]See legend	N/A				

Ref.	*Con.*	*Sample*	*Diag.*	*Mean age*	*PMI*	*Brain areas*	*Methodology*	*Main results*	*Brain Bank*
**Schnieder, T et al. 2019**	NSD	n = 11<br>F = 5<br>M = 5	N/A	55	15	dPFWM<br>vPFWM	PB formalin and paraffin.<br>CD163 and GLUT1 IHC.<br>Stereological characterization of vessel density.<br>HE staining.	No significant effects of SD on the fraction of vascular surface area immunoreactive for CD163.	NYSPI
	SD	n = 25<br>F = 12<br>M = 13	AD or SCZ	56	12				
**Zhang, L et al. 2020**	CT	n = 34<br>F = 9<br>M = 25	N/A	45	29	DLPFC<br>ACC	Frozen tissue.<br>RT-PCR of ALDH1L1, GFAP, GLT1, GS, S100b, CD68, CX3CR1, HLA-DR, IBA1, P2RY12, TREM2, TSPO, MBP, MOG, OLIG2 and PLP1.	Higher ALDH1L1 in both the DLPFC and ACC in individuals with SCZ *versus* CT, particularly, in NSD.<br>No differences in DLPFC for CD68, CX3CR1, HLA-DR, IBA1, P2RY12, TREM2, TSPO expression.<br>Increased CX3CR1, P2RY12, and TREM2 in the ACC of SD compared to NSD.	SFNC
	SD	n = 7<br>F = 3<br>M = 4	SCZ	39	27				
	NSD	n = 28<br>F = 6<br>M = 22	SCZ	44	30				
**Snijders, G et al. 2020**	CT	n = 27<br>F = 17<br>M = 10	N/A	81	7	MFG<br>STG	CD11B-positive cell isolation from fresh post-mortem tissue.<br>Flow cytometry (CD45, CD11b, CD11c, CD14, CD16, CD32, CD40, CD64, CX3CR1,	Increase of TMEM119 and CX3CR1 mRNA levels, and a decrease in CD163 protein expression between individuals	NBB

Ref.	*Con.*	*Sample*	*Diag.*	*Mean age*	*PMI*	*Brain areas*	*Methodology*	*Main results*	*Brain Bank*
	SD	n = 1[h]	MDD	See legend[h]	See legend[h]	TLM SZ	HLA-DR, CD163, CD172α, CD200R, MRC1, CD83, and CD86). qPCR (TMEM119, CX3CR1, IL1β, IL6, CD163 and MRC1). Stimulation of cells with LPS or DEX.	with MDD and CT. No difference in the expression of HLADR, IL1β, and IL6 or in the response to LPS and DEX. No specific differences detected for the one suicide case present in the cohort with MDD.	
**Zeng, D et al. 2020**	CT	n = 29 F = 6 M = 23	N/A	44	13	DLPFC	Weighted correlation network analysis on previously published RNA-seq data. GO and pathway enrichment analysis, protein-protein interaction prediction. Genes obtained were investigated in previous RNA-seq data from DG and CA1 and in published methylation data.	GOs most correlated with SD and MDD involved in cell death and apoptosis enriched in endothelial and microglial cells. The top 10 genes implicated in SD include FOSB, DUSP1, NFKBIA, TLR2, NR4A1. 92 genes had at least one highly significant differentially methylated positions between CT and MDD SD.	DMIN
	SD	n = 21 F = 8 M = 13	MDD	52	16				
	NSD	n = 9 F = 3 M = 6	MDD	58	15				
**Petrasch-Parwez, E et al. 2020**	CT	n = 17 F = 4 M = 13	N/A	45	29	aMCC	PB formalin and cryostat. IBA1 IHC. Cresyl-violet staining. Qualitative morphological analysis (undulated or extended processes) based on staining. Monocytes localized within blood vessels	No difference in cell density between CT and SD. Increased density of IBA1-positive cells in the right compared to left aMCC in individuals with SCZ and BD. Increased density of IBA1-positive cells in individuals with	SFNC
	SD	n = 9 F = 5 M = 4	BD or SCZ	42	32				

Ref.	*Con.*	*Sample*	*Diag.*	*Mean age*	*PMI*	*Brain areas*	*Methodology*	*Main results*	*Brain Bank*
	NSD	N =17<br>F = 4<br>M =13	BD or SCZ	47	33		were excluded. Only cells located in the parenchyma were evaluated.	BD<br>who died by SD, compared to NSD.<br>No particular effect of SD on qualitative microglial morphology.	
**Glavan, D et al., 2021**	CT	n = 7<br>F = 3<br>M = 4	N/A	42	15	HIP<br>AMY<br>TLM<br>PFC	Microarray and GO analyses.<br>RTqPCR, small nucleolar RNA, miRNA quantification.	Downregulation of pathways involved in the immune response in the TLM and AMY in SD.<br>Upregulation of pathways involved in neuronal death and downregulation of pathways involved in synaptic plasticity in the HIP of SD.<br>Upregulation of pathways involved in synaptic plasticity and oxidative stress in the TLM of SD.<br>44 genes differentially regulated between CT and SD, including increased transcripts of CX3CR1 and C3 in AMY, HIP and TLM.	DBCBB
	SD	n = 20<br>F = 9<br>M = 11	MDD[I]	39	28				
**Zhang, L et al., 2021**	CT	n = 12<br>F = 4<br>M = 8	N/A	47	25	DLPFC<br>ACC	qPCR of ALDH1L1, GFAP, S100b, CD11B, CD45, CX3CR1, HLA-DR, IBA1,	Upregulation of CD68 and TSPO in the DLPFC of MDD, with a SD effect on CD68 and a natural death effect on TSPO.	SMRI

Ref.	Con.	Sample	Diag.	Mean age	PMI	Brain areas	Methodology	Main results	Brain Bank
	SD	n = 17<br>F = 7<br>M =10	MDD	40	30		TMEM119, TREM2, P2RY12, CD68, TSPO, MBP, MOG, OLIG2, PLP1	CD11B and P2RY12 reduced in the ACC of non-suicidal deaths with MDD, but not in SD compared to CT.	

Supplementary Table 1. Studies investigating microglial properties or functions in SB. Summary of research studies found in the literature that contain data on microglial properties in relation to suicide behaviors. Mean age represented in years and Post-mortem interval (PMI) in hours. Abbreviations are listed in order of appearance in the table: reference (Ref.), conditions (Con.), diagnoses (Diag.), Controls (CT), suicide deaths (SD) female (F), male (M), not applicable (N/A), schizophrenia (SCZ), Anterior cingulate cortex (ACC), dorsolateral prefrontal cortex (DLPFC), hippocampus (HIP), mediodorsal thalamic nucleus (MTN), phosphate-buffered (PB), Human leukocyte antigen- DR (HLA-DR), immunohistochemistry (IHC), Magdeburg Brain Collection (MBC), non-suicide death (NSD), major depressive disorder (MDD), bipolar disorder (BD), Anterior midcingulate cortex (aMCC), subgenual ACC (sACC) and Pregenual ACC, (pACC), quinolinic acid (QUIN), , dorsal anterior cingulate cortex (dACC), ionized calcium binding adaptor molecule 1 (IBA1), protein tyrosine phosphatase receptor type C (CD45), CD68 molecule (CD68), C-C motif chemokine ligand 2 (MCP1 or CCL2), selectin E (SE), catenin alpha 1(CateninA1), tight junction protein 1 (ZO1), interleukin 1 beta (IL1β), interleukin 10 (IL10), tumor necrosis factor (TNF), interleukin 1 receptor antagonist (1L1RA), glyceraldehyde-3-phosphate dehydrogenase (GAPDH), Douglas-Bell Canada Brain Bank (DBCBB), affective disorders (AD), dorsal Prefrontal white matter (dPFWM), ventral Prefrontal white matter (vPFWM), Macedonian/New York State Psychiatric Institute (NYSPI), Hippocampal *cornu ammonis* (CA) 1, dentate gyrus (DG), non-suicide asphyxiation death (NSAD), asphyxiation suicide-death (ASD), hematoxylin and eosin (HE), cytochrome b-245 beta chain (NOX2), 8-8-hydroxy-2' - deoxyguanosine (8-OHdG), RNA binding fox-1 homolog 3 (NEUN), glial fibrillary acidic protein (GFAP), S100 calcium binding protein A9 (MAC387), S100 calcium binding protein B (S100b), glutamate decarboxylase 67 (GAD67), solute carrier family 17 member 7 (VGLUT1), dopamine transporter (DT1), Section of Legal Medicine (SLM), ventrolateral prefrontal cortex (VLPFC), indoleamine 2,3-dioxygenase 1 (IDO), tryptophan 2,3-dioxygenase (TDO2), interferon gamma (IFNγ), prostaglandin-endoperoxide synthase 2 (COX2), kynurenine (KYNA), 3-hydroxykynurenine (3-HK), tryptophan (TRYP), kynurenine (KRNA), Clinical Brain Disorders Branch (CBDB), gene ontology (GO), lipopolysaccharide (LPS), RNA-sequencing (RNA-Seq), microRNA (miRNA), cannabinoid receptor 2 (CB2), G protein-coupled receptor 55 (GPR55), immunofluorescence (IFL), Institute of Legal Medicine (ILM), suicidal thinking (ST), no suicidal thinking (NST), prefrontal cortex (PFC), translocator protein (TSPO), positron emission tomography (PET), C-reactive protein (CRP), CD163 molecule (CD163), solute carrier family 2 member 1 (GLUT1), aldehyde dehydrogenase 1 family member L1 (ALDH1L1), glutamate transporter 1 (GLT1), glutamine synthetase (GS), C-X3-C motif chemokine receptor 1 (CX3CR1), purinergic receptor P2Y12 (P2RY12), triggering receptor expressed on myeloid cells 2 (TREM2), myelin basic protein (MBP), myelin oligodendrocyte glycoprotein (MOG), oligodendrocyte transcription factor 2 (OLIG2), proteolipid protein 1 (PLP1), Stanley Foundation Neuropathology Consortium (SFNC), medial frontal gyrus (MFG), superior temporal gyrus (STG), thalamus (TLM), subventricular zone (SZ), integrin subunit alpha M (CD11B), integrin subunit alpha X (CD11c), CD14 molecule (CD14), Fc gamma receptor IIIa (CD16), Fc gamma receptor IIb (CD32), CD40 molecule (CD40), Fc gamma receptor Ia (CD64), signal regulatory protein alpha (CD172α), CD200 receptor 1 (CD200R), CD83 molecule (CD83), CD86 molecule (CD86), transmembrane protein 119 (TMEM119), mannose receptor C-type 1 (MRC1), dexamethasone (DEX), Netherlands Brain Bank (NBB), FosB proto-oncogene, AP-1 transcription factor subunit (FOSB), dual specificity phosphatase 1

(DUSP1), NFKB inhibitor alpha (NFKBIA), toll like receptor 2 (TLR2), nuclear receptor subfamily 4 group A member 1 (NR4A1), Real time-quantitative polymerase chain reaction (RT-qPCR), amygdala (AMY), Stanley Medical Research Institute (SMRI). [a]Males were younger than females, [b]Trend towards an older age of the CT compared to SD, [c]Delirium, behavior disorders, aspecific psychiatric disorder, alcohol addiction, [d]Mean age not available, [e]No data on sex, age, or PMI for SD cohort, [f]No data on sex for CT, [g]No data on sex or age for ST cohort, [h]No data on sex, age or PMI for SD.
